# Is percutaneous nephrolithotripsy feasible in ipsilateral lumbar incisional hernia? A report of two patients

**DOI:** 10.1093/jscr/rjae456

**Published:** 2024-07-11

**Authors:** Anh Toan Do, Huynh Dang Khoa Nguyen, Ngoc Thai Nguyen

**Affiliations:** Department of Urology, Faculty of Medicine, University of Medicine and Pharmacy at Ho Chi Minh City, 217 Hong Bang Street, Ward 11, District 5, Ho Chi Minh City, 70000, Vietnam; Binh Dan Hospital, 371 Dien Bien Phu Street, Ward 4, District 3, Ho Chi Minh City, 70000, Vietnam; Department of Urology, Faculty of Medicine, University of Medicine and Pharmacy at Ho Chi Minh City, 217 Hong Bang Street, Ward 11, District 5, Ho Chi Minh City, 70000, Vietnam; Department of Urology, Faculty of Medicine, University of Medicine and Pharmacy at Ho Chi Minh City, 217 Hong Bang Street, Ward 11, District 5, Ho Chi Minh City, 70000, Vietnam; Binh Dan Hospital, 371 Dien Bien Phu Street, Ward 4, District 3, Ho Chi Minh City, 70000, Vietnam

**Keywords:** incisional hernia, percutaneous nephrolithotripsy, recurrent kidney stones

## Abstract

Incisional hernia refers to an abdominal wall defect at the site of a previous surgical incision. In this paper, we describe two patients who previously underwent open kidney stone surgery several years ago and had the ipsilateral recurrent stones. They were both managed by a mini percutaneous nephrolithotripsy (PCNL) to treat kidney stones. Case 1 was a 50-year-old female with right recurrent staghorn stones after 5 years of open surgery and required two PCNL procedures to achieve stone-free status. Case 2 was a 74-year-old male with significant comorbidities who had a right 27 mm recurrent kidney stone after 10 years of open nephrolithotomy. Both patients experienced no postoperative complications after PCNL. These cases show that in cases of lumbar incisional scar hernias, mini PCNL with ultrasound guidance and proper patient positioning can be an optimal approach for kidney stone treatment.

## Introduction

In the modern era, the minimally invasive technique is evolving to achieve both effectiveness and safety in patient health care. Regarding urolithiasis management, Fernström and Johansson described the first percutaneous nephrolithotripsy (PCNL) in 1976 [1]. Since then, PCNL has become the standard option for large and complex kidney calculi, replacing open surgery in the vast majority of patients [1–3].

However, stone recurrence is common after primary treatment. In some cases, with previous flank incisional scars from an open lithotomy, surgeons may encounter difficult scenarios during PCNL. In this report, we describe PCNL in two incisional hernia patients with a history of open nephrolithotomy and ipsilateral recurrent kidney stones.

## Case presentation

### Case 1

**Figure 1:**
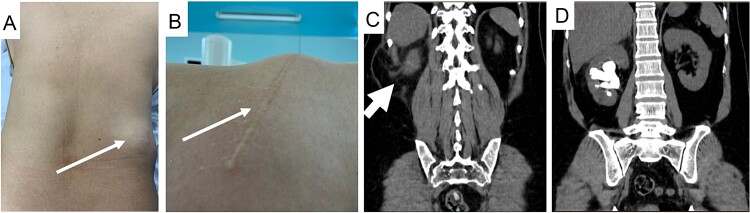
Images of the patient with right flank incision and hernia bulge with clinical picture (A and B, thin arrow), hernia sac on CT scan (C, thick arrow), and staghorn stone (D).

A 50-year-old female presented with a progressively enlarging right lumbar bulge persisting for 3 years following previous open surgery to treat a large staghorn calculus in her right kidney. Examination revealed a visible flank scar and hernia bulge in the supine position (Fig. 1A and B), confirmed by an abdominal computed tomography (CT) scan (Fig. 1C). The CT scan also revealed bowel contents within the sac and a recurrent staghorn right kidney stone (Fig. 1D).

**Figure 2:**
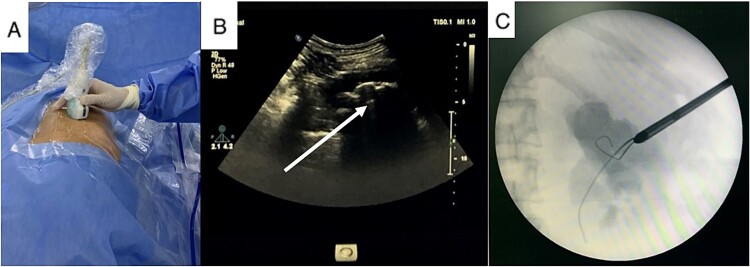
US guidance with carefully exclude bowel during renal puncture (A), real-time US showed the stone and its acoustic shadow (B, arrow), and tract dilation using safety guidewire on fluoroscopy monitor (C).

After a thorough assessment, we decided to treat her renal calculi first and deferred treatment of her incisional hernia. A prone mini PCNL was performed using combined ultrasound (US) and fluoroscopy guidance for renal puncture (Fig. 2). After the first session, significant residual stone remained, leading us to perform the salvage PCNL through the original nephrostomy tract and achieve a stone-free result on the C-arm screen. She was discharged on postoperative Day 3 without any complication.

### Case 2

**Figure 3:**
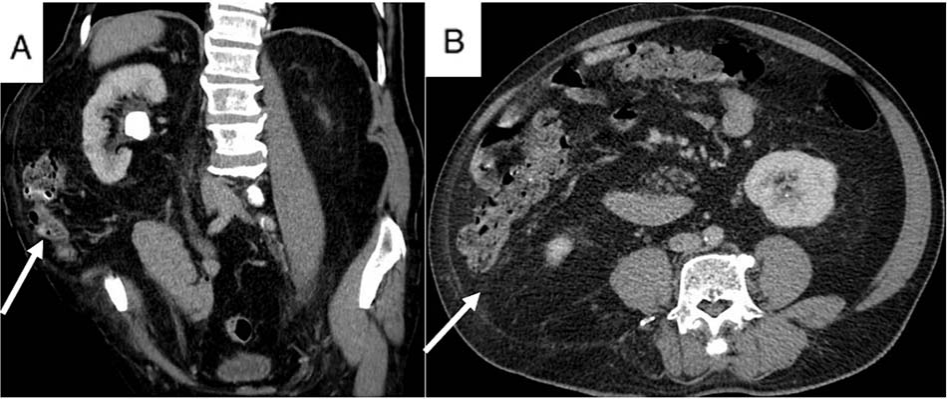
Patient’s CT scan shows right UPJ stone (A) and lumbar hernia with mesentery inside (A and B, arrow).

A 74-year-old male presented to our hospital with persistent right flank pain for 5 months who had undergone previous open nephrolithotomy to treat a staghorn stone 10 years ago. He was classified ASA class III due to moderate heart failure, characterized by a 40% ejection fraction. Physical examination revealed a bulky mass on the right lumbar region lateral to the anterior axillary line, consistent with CT scan findings (Fig. 3). A 27 mm stone was observed in the right ureteropelvic junction (UPJ).

**Figure 4:**
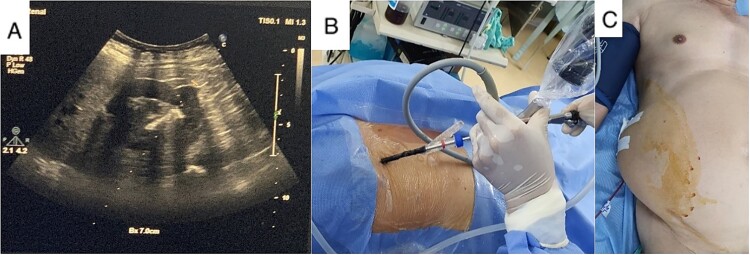
Real-time US guide for renal puncture (A), mini PCNL with lateral position (B), and nephrostomy after completing stone clearance (C).

Baseline preoperative laboratory tests were normal. A lateral flank position mini PCNL was performed using combined US and flouroscopy guidance for renal puncture (Fig. 4A). After 35 min of lithotripsy, the stone burden was successfully removed, and the patient was discharged after 3 days without any complications. He declined treatment for his lumbar bulge after considering the risks associated with another operation.

## Discussion

The aim of treating kidney calculi is to remove all stones while minimizing renal function loss and reducing surgical complications [4]. According to recent guidelines, PCNL has become the standard option for removing renal stones larger than 2 cm, offering equivalent stone-free rates with lower morbidity, fewer complications, and smaller scars [2, 3].

An incisional hernia is defined as any abdominal wall gap with or without a bulge in the area of a postoperative scar, found by clinical examination or imaging [5]. The incidence has been observed in 0.4%–17% of patients who have undergone flank incisions. Risk factors include a high BMI, smoking, diabetes, hypertension, chronic obstructive pulmonary disease, and end-stage renal disease [6, 7]. Current studies indicate intervention only if the hernia causes symptoms, such as pain, digestive disorders, aesthetic concerns, or psychological impact on the patient [6, 8]. In the first patient, we decided to treat the kidney stones first because the patient preferred to undergo kidney stone treatment with minimal invasion and would address the hernia afterward. In the second case, the patient had multiple comorbidities and complained primarily of lower back pain, likely due to a kidney stone at the UPJ location, and had no desire to treat the hernia.

In cases of lumbar incisional hernias, there is a higher risk of iatrogenic injury to the contents of the hernia sac, especially involving the bowel and mesentery, during PCNL. Therefore, a preoperative CT scan of the kidney is essential to confirm the diagnosis and identify the structures of the hernia bulge. Intraoperative US allows real-time simultaneous tracking of the puncture needles, thus aiding in the avoidance of inadvertent injuries to adjacent organs [4, 9]. In these two cases, real-time US imaging is particularly helpful for puncture, especially in cases involving hernias, to prevent bowel injury. At times, we consulted with radiologists to ensure optimal access tracts.

Moreover, the patient’s positioning in special situations also plays an important role in safely performing the procedure. In the first case, the patient was placed in the prone position to achieve a wider working space, reducing the risk of iatrogenic injuries. However, due to compromised cardiovascular function in the second case, we opted to perform mini PCNL in the lateral position to minimize hemodynamic risks [1]. There is limited data regarding the safety and efficacy of PCNL in patients with ipsilateral lumbar hernias. In 2017, Adam reported a case of mini PCNL for a 22-mm renal stone in a 49-year-old man with an incisional hernia and achieved stone-free status after a single procedure [10]. In the first case, the patient required a second look at PCNL due to the staghorn stone, while in the second case involving simple calculi, only one session was needed to achieve stone-free status. There were no postoperative complications in either case, demonstrating that mini PCNL is a viable option for managing recurrent stones in patients with incisional hernias.

## Conclusion

In the modern era, less invasive techniques have become standard treatments for surgical management. Although there is limited existing literature on PCNL in cases of incisional hernia, the initial outcomes of this approach have been favorable. US guidance is necessary to ensure puncture accuracy and to avoid complications during renal access. Further evaluation is required to assess the role of PCNL for stone removal in patients with lumbar hernias.

## Data Availability

All relevant data that supports the findings of this study is included in the article.
