# Correction to: Is percutaneous nephrolithotripsy feasible in ipsilateral lumbar incisional hernia? A report of two patients

**DOI:** 10.1093/jscr/rjag240

**Published:** 2026-04-25

This is a correction to: Anh Toan Do, Huynh Dang Khoa Nguyen, Ngoc Thai Nguyen, Is percutaneous nephrolithotripsy feasible in ipsilateral lumbar incisional hernia? A report of two patients, *Journal of Surgical Case Reports*, Volume 2024, Issue 7, July 2024, rjae456, https://doi.org/10.1093/jscr/rjae456

A new respective reference should have been included at the end of the **References** list: “11. Do, A. T., Nguyen, N. T., Nguyen, D. T., et al (2025). Case report: mini PCNL in large renal stone and ipsilateral flank hernia due to previous open surgery. JCMHCH, (81), 47–50. https://doi.org/10.38103/jcmhch.81-7.”

In the section **Case Presentation**, the following should have been added to the beginning of the first paragraph of **Case 1**: “The clinical details of this case were previously reported in a preliminary local publication [11]. However, the time since the previous open surgery and the length of postoperative hospital stay have been updated in the present report to accurately reflect the actual clinical records.”

The end of the caption to Figure 1 should have the following sentence added: “Figure 1B is reprinted with permission from [11].”

The changes have been outlined only in this correction notice to preserve the version of record.

